# INSIGHT: Combining Fixation Visualisations and Residual Neural Networks for Dyslexia Classification From Eye‐Tracking Data

**DOI:** 10.1002/dys.1801

**Published:** 2025-01-22

**Authors:** Roman Svaricek, Nicol Dostalova, Jan Sedmidubsky, Andrej Cernek

**Affiliations:** ^1^ Department of Educational Sciences, Faculty of Arts Masaryk University Brno Czech Republic; ^2^ Department of Machine Learning and Data Processing, Faculty of Informatics Masaryk University Brno Czech Republic

**Keywords:** AI‐based diagnosis, deep learning, dyslexia, eye movement, eye tracking, fixation data classification, ResNet18

## Abstract

Current diagnostic methods for dyslexia primarily rely on traditional paper‐and‐pencil tasks. Advanced technological approaches, including eye‐tracking and artificial intelligence (AI), offer enhanced diagnostic capabilities. In this paper, we bridge the gap between scientific and diagnostic concepts by proposing a novel dyslexia detection method, called INSIGHT, which combines a visualisation phase and a neural network‐based classification phase. The first phase involves transforming eye‐tracking fixation data into 2D visualisations called Fix‐images, which clearly depict reading difficulties. The second phase utilises the ResNet18 convolutional neural network for classifying these images. The INSIGHT method was tested on 35 child participants (13 dyslexic and 22 control readers) using three text‐reading tasks, achieving a highest accuracy of 86.65%. Additionally, we cross‐tested the method on an independent dataset of Danish readers, confirming the robustness and generalizability of our approach with a notable accuracy of 86.11%. This innovative approach not only provides detailed insight into eye movement patterns when reading but also offers a robust framework for the early and accurate diagnosis of dyslexia, supporting the potential for more personalised and effective interventions.

## 1 Introduction

Developmental dyslexia is a specific learning disorder of neurobiological origin (Shaywitz et al. 2001) that has received significant attention in recent decades. Dyslexia affects roughly 5%–17.5% of the global population (see e.g., Habib 2000; Yang et al. 2022) it is estimated to affect 5%–8% in the Czech Republic (Caravolas, Mikulajová, and Kucharská 2019). Dyslexia appears as errors in spelling and difficulties with decoding letters and words, even though the reader's intellectual level and motivation are both at an average or above‐average level (Lyon, Shaywitz, and Shaywitz 2003). Dyslexic symptoms may result in difficulties with reading comprehension (Peterson and Pennington 2012) and a subsequent reduced motivation to read (Gabrieli 2009). Dyslexia usually emerges in the early school years when children are first exposed to written text. In that period, initial difficulties with reading at the level of letters and whole words or sentences begin to manifest (Gabrieli 2009). If these symptoms are not resolved, young readers may develop severe complications in the form of reduced emotional and social development and unsatisfactory performance within the school environment (Livingston, Siegel, and Ribary 2018). For these reasons, it is crucial to pay increased attention to high‐quality, precise, and objective diagnostics for dyslexia that can ensure a detailed understanding of dyslexic difficulties in a given individual and would subsequently support the areas of reading and reading literacy at the age when learning to read is a crucial step.

When describing the symptoms of dyslexia, it is crucial to also introduce the language based aspects of its manifestations. The Czech language belongs to the family of the West‐Slavic alphabetic languages with a relatively consistent orthographic system, which may, however, contain some irregularities in phoneme‐grapheme transparency, which may subsequently affect the spelling of the individual when learning to read. Czech words are often polysyllabic, based on open syllables, but complex syllables composed of multiple consonants are also relatively common (Caravolas, Mikulajová, and Kucharská 2019).

As in other languages, dyslexia in Czech is manifested mainly by slow and dysfluent reading. Complications may also occur during the reading of phonologically complex words based on longer consonant chunks. In the context of reading, double reading and persistent difficulties with text comprehension often occur, which may be related to grammatical and syntactical phenomena in Czech (e.g., free sentence order if compared to English) (Caravolas, Mikulajová, and Kucharská 2019).

Dyslexia is currently diagnosed in the Czech Republic most often at the age of 8–10 years old (Caravolas, Mikulajová, and Kucharská 2019), that is, when the school child should already have full control of their reading skills and be able to receive new knowledge as part of the educational process. The diagnosis of specific learning disabilities, including dyslexia, is usually made using standardised test batteries in pencil‐and‐paper versions, during which the test subject completes a series of verbal and non‐verbal tasks aimed at testing reading competences (reading a standard text or pseudo‐text) and phonological skills (e.g., analysis and synthesis of words) and the quality of visual perception. The diagnostic battery also includes writing and orthographic evaluation (Bednářová 2015). For a diagnosis, a psychologist also focuses on the child's behaviour and expressions throughout the entire testing process (Caravolas, Mikulajová, and Kucharská 2019). Current methods for diagnosing dyslexia focus primarily on the test subject's cognitive abilities. The assessment of diagnostic tasks is based only on the child's performance (e.g., reading aloud, error rate). Within the diagnostic process itself, the diagnostician is not able to observe and subsequently analyse what exactly the child observes when solving test tasks, since the registration of eye movements is not included in the standard diagnostic process. Additionally, the diagnosis is highly dependent on the subjective assessment of a psychologist. These assessment methods are not able to capture reading, which is a cognitive process, in its natural form (Nilsson Benfatto et al. 2016). In general, reading is a process that can be analysed by objective measurements, including gaze recordings. Current research in reading has shown significant differences in the gaze patterns of dyslexic readers (Rayner 1998). Therefore, a precise screening method with gaze parameters for enhancing the screening objectivity is essential.

Research in the field of dyslexia and the reading process is, among other topics, devoted to the recordings of eye movements. Several theories have tried to shed light on the origins of dyslexia (see phonological theory, Ramus 2001; Snowling 1998); some theories, including the magnocellular theory (see Stein 2001) and the cerebellar theory (Nicolson, Fawcett, and Dean 2001), are closely related to eye movements. There have been rich discussions around the origins and causes of dyslexia (see Vellutino et al. 2004). More recent studies have clarified that, in terms of the magnocellular theory, specific eye‐movement patterns are not causally related, but rather correlated (Hutzler et al. 2006). Nevertheless, the eye movements of people with dyslexia differ from the eye movements of non‐dyslexic readers while reading words or different types of text, and they also have different ways of reading. According to Rayner (1998), readers with dyslexia generally show longer fixation durations, more fixations, shorter saccades and more regressive saccades than non‐dyslexic readers. Hutzler and Wimmer (2004) added that as word length increases, so does the number of fixations for dyslexic readers. Research has indicated that if a dyslexic child reads a text that is intended for their age group, they show dyslexic symptoms. However, if dyslexic readers read a text intended for a lower age group, their typical dyslexic gaze patterns in reading (e.g., reading speed, number of saccades and regressions) tend to occur less frequently (Trauzettel‐Klosinski et al. 2010, see also Ciuffreda and Tannen 1995; Smyrnakis et al. 2017). When reading pseudo‐words, dyslexic readers show a higher fixation count and also longer fixation duration than non‐dyslexic readers (Hutzler and Wimmer 2004). Increasing pseudo‐word length negatively affected fixation count in dyslexic readers (Hutzler and Wimmer 2004).

In general, the main gaze patterns for the analysis of eye movements in regular eye‐tracking research are fixations and saccades. Fixation is the state when the eye is relatively still for a certain period of time (tens of milliseconds up to a few seconds) (Holmqvist et al. 2015). Three micro‐eye movements are performed during fixation: microsaccades, drifts and tremor for optimization of visual processing (Martinez‐Conde, Macknik, and Hubel 2004). When fixating, overall visual perception and visual information uptake is being processed (Martinez‐Conde 2006). Saccades are fast eye movements from one fixation to another (Holmqvist et al. 2015). Saccades have great informative value, with the most common parameters being the number of forward and backward saccades or their duration and amplitude. The analysis of saccadic values can provide information about a reader's strategy and ability to navigate words and text (Rayner 1998). It is evident that the gaze differences between dyslexic and non‐dyslexic readers are considerable. Eye movement patterns are usually analysed in order to evaluate the statistical significance of these metrics. Recent research (see e.g., Smyrnakis et al. 2017; Nilsson Benfatto et al. 2016) has presented some attempts to use deeper analyses with artificial intelligence (AI) approaches and their potential to classify and predict dyslexic patterns.

The literature varies in what specific gaze‐event characteristics are the most useful for dyslexia classification. The selected characteristics are then classified using different AI methods, usually based on support vector machines (SVMs), *k*‐Nearest Neighbour (*k‐*NN), or random forest classifiers. However, these traditional classification methods are becoming obsolete due to the success of artificial neural networks and deep learning, which have gained a lot of attention in the computer science field (Goodfellow, Bengio, and Courville 2016). Artificial neural networks are machine learning techniques inspired by the biological neural networks that constitute the brain. They are collections of smaller units, called (artificial) neurons, connected to pass a signal in the form of real numbers. Individual variants differ in either the architecture or the unit design. Specifically, fixed‐size characteristics (e.g., 100‐D real‐value vectors of gaze‐event features) can be classified by multilayer perceptrons (MLPs), variable time‐series (e.g., the evolution of the center of captured fixations in time) by recurrent neural networks (RNN), or images by convolutional neural networks (CNNs). The advantage of such neural networks is that they can learn complex non‐linear dependencies from input data and thus generally achieve high accuracy in classification tasks. The disadvantage is that they require large amounts of training data: the ResNet18 CNN (He et al. 2016), capable of classifying general‐purpose photos into a 1000 predefined categories, was trained on a million photo examples.

### 1.1 Related Work

Current research presents attempts to use gaze patterns to create automated tools based on AI approaches for screening and classifying dyslexia, which may lead to precise and objective measurements when screening and diagnosing dyslexia. One of the first attempts, by Rello and Ballesteros (2015), aimed at a prediction model for dyslexic readers based on eye‐movement recordings. For this purpose, they used a remote eye‐tracker to measure 97 subjects (48 dyslexic) in the age range of 11–54 years old, while reading 12 different texts, resulting in a total of 1135 readings after the dataset cleaning. Number, mean and sum of visits, and number, mean and sum of fixations were considered for SVMs as a machine learning method, reaching an accuracy of 80.18%.

Nilsson Benfatto et al. (2016) used another approach, considering 185 children (97 children considered high risk for dyslexia) in a specific age range of 9–10 years old with the main aim of creating a fast and accurate screening method. Eye‐tracking data were gathered with a goggle eye‐tracker and reading one paper‐printed text as a main stimulus. They used a linear SVM and the recursive feature elimination as a feature selection method, showing a total of 48 features (saccade and fixation parameters) and resulting in 95.6% accuracy. This dataset was re‐used by Nerušil et al. (2021) with a CNN approach achieving 96.6% accuracy and by Jothi Prabha and Bhargavi (2020) showing 95.6% accuracy with a hybrid SVM—Particle Swarm Optimization (SVM‐PSO) model based on fixation (average number of fixation and fixation duration) and saccadic (number of saccades and saccade duration) features. In a study based on the same dataset, Jothi Prabha, Bhargavi, and Deepa Rani (2023) presented a clustering method based on *k*‐means for classifying high (severe) and low dyslexic performance.

Smyrnakis et al. (2017) presented a fast and objective method focusing on eye‐movement patterns in dyslexic children aged 8.5–12.5 in Greece. A total of 69 participants underwent eye‐tracking measurements based on silent reading of texts and subsequent comprehension questions. With an eye‐tracker set up at a sampling rate of 60 Hz, the following gaze parameters were selected for further analysis: fixation duration, saccade length, short regressive saccades and the total fixation count when reading the text. The maximum classification accuracy was reached with the TRS method, resulting in 94.2% correct classification. Smyrnakis et al. (2017) used an easier version of another text, resulting in 87.9% classification accuracy with the same procedure. A follow‐up study by Asvestopoulou et al. (2019) using the dataset from Smyrnakis et al. (2017) was applied to new machine learning approaches, such as SVM, *k*‐means and the Naive Bayes classifier and employed a reduced number of gaze parameters.

Screening for dyslexia using machine learning was also approached by Raatikainen et al. (2021) using eye‐tracking data from 161 students with an average age of 12.5 years, 30 of whom had dyslexia. For the machine learning analysis, Random Forest was used to extract crucial eye‐tracking features which were then analysed using SVM based on which they achieved an accuracy of 89.7%.

Vajs et al. (2022) gathered another dataset in Serbian, consisting of 30 dyslexic and control subjects aged 7–13. Gaze data were collected on a sampling rate of 120 Hz and nine gaze metrics were considered for the classification, including fixation count, duration and frequency, and saccade count, duration and frequency. Vajs et al. (2022) used logistic regression, SVM, *k*‐NN and random forest and reached almost 94% accuracy.

It is evident that the use of gaze tracking and AI‐based approaches for the purpose of dyslexia detection has evolved intensively in the last decade. However, the existing research exhibits considerable inconsistency in terms of linguistic, methodological and technological factors. Therefore, there is a compelling need for a comprehensive investigation. Furthermore, the studies currently published mainly focus on testing fundamental machine learning and AI techniques to reach high classifying accuracy based on gaze measures. However, the majority of the eye‐tracking data quality is insufficient for conducting thorough reading analyses due to the inadequate sampling rate of eye‐tracking devices used. Additionally, the present research lacks simplified visual representations of eye‐tracking data that would facilitate fast, precise and high‐quality screening and provide further insights into reading disabilities for the experts. In our study, we propose a rapid screening assessment of gaze data through fixation visualisations and employ a neural network approach to evaluate overall reader performance.

### 1.2 Aims of the Paper

In this paper, our goal is to present INSIGHT, a novel AI‐based system for dyslexia classification, which we evaluate on a homogeneous sample of dyslexic and control participants on three types of text‐reading tasks.

In particular, we propose a new concept of a fixation image, a 2D image representation of eye‐tracking fixations. This visual representation allows diagnosticians of reading disability to easily interpret eye movements and, more importantly, enables the straightforward application of CNNs that are very effective in the image classification task. Additionally, we tested the generalizability of proposed AI‐based methods on a publicly available foreign dataset that provides eye‐tracking features of dyslexic and non‐dyslexic readers.

## 2 Methods of Experiment

### 2.1 Participants

We measured 35 elementary school pupils, aged 9–10 years (*M* = 9.11, SD = 0.31), including 15 females and 20 males. Participant recruitment was realised with the help of the Psychological Counselling Centre and collaborating elementary schools in (Anonymized city). Based on Caravolas, Mikulajová, and Kucharská (2019), we decided to focus on primary school pupils aged 9–10 who had been diagnosed with dyslexia as appropriate participants for the experimental group (*N = 13*). The control group consisted of primary school pupils aged 9–10 who did not show any symptoms of dyslexia (*N = 22*). Data collection was performed in August and September 2021. The whole procedure took approximately 40 min, and the legal representatives of the children received 200 CZK as a reward. We sought written consent from all of the parents of the children. Parents were assured of confidentiality and of the freedom to withdraw at any time. All participants were assigned pseudonyms, and any personally identifying information was removed from the data prior to analysis. The Research Ethics Committee of (Anonymized) University approved the research plan and its implementation.

### 2.2 Stimuli

To investigate the differences and develop classification models, three reading tasks were designed. Research (e.g., Trauzettel‐Klosinski et al. 2010; Smyrnakis et al. 2017) has indicated that if a child with dyslexia reads a text that is intended for their age group, they show dyslexic symptoms. However, when some children with dyslexia read a text intended for a lower age group, their dyslexic gaze patterns tend to occur less frequently. For these purposes, three different reading stimuli were prepared that were thematically non‐related. All three text stimuli (categorised subsequently as at‐level text, below‐level text and pseudo‐text) were adopted from the standardised battery for diagnosing dyslexia in the Czech Republic (Bednářová 2015). Additionally, we adjusted the texts for appropriate eye‐tracking measurement (letter size and line spacing according to the visual angle). For detailed descriptions of each text's parameters, see Table 1. After each text, two sentences related to the previous text stimuli appeared. However, these sentences are not used in this study in any way.

**TABLE 1:** Summary of the main parameters of each text presented to the participants.

	Number of lines	Number of sentences	Number of words	Average number of words per line
At‐level text	11	11	166	15.09
Below‐level text	8	23	113	14.13
Pseudo‐text	8	24	139	17.38

**FIGURE 1:**
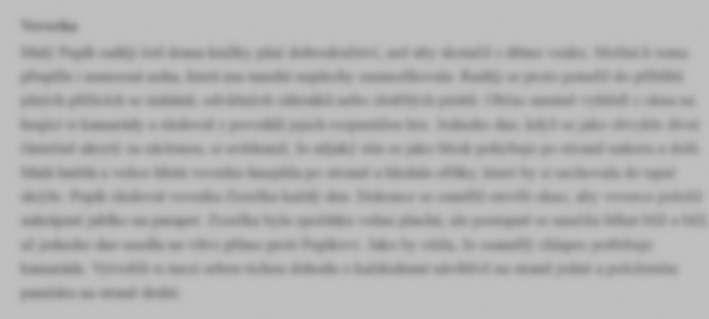
Example of at‐level text stimulus (blurred version). The stimulus is blurred for text content protection (see Bednářová 2015). The stimulus in Figure 1 has been trimmed for purposes of visualisations and further analyses.

At‐level text was implemented as a core same‐age‐level text for dyslexia detection. The at‐level text was originally developed for diagnosing developmental dyslexia in the 3rd and 4th grades of elementary school. As we were focusing only on the age group of 9–10 years old, we considered this text relevant as a same‐age‐level text. Thematically, the text described a story about a boy watching a squirrel from a window at home. The original text is in a paper‐and‐pencil version; we tried to retain the exact font, letter size and lining for the eye‐tracking stimulus. All three text stimuli were shown to the participants for only 2 min following the original diagnosing instructions. However, the text was shortened due to the screen size. The text was written with black letters on a grey background and was positioned in the center of the screen. An example of the stimulus is presented in Figure 1.

Below‐level text was added to observe dyslexic and non‐dyslexic reading aspects when processing less‐demanding text. The below‐level text was also adopted from the diagnostics test battery (Bednářová 2015). The text is usually used for diagnosing second graders (i.e., 7–8 years old). Thematically, this stimulus describes a short family story of a young boy named Bert. We retained the original text specifications, such as font, size and lining. However, the below‐level text was displayed with a larger size and lining in the original version, so we followed these specifications by recalculating the proper visual angle.

Pseudo‐text was also retaken from the diagnostic test battery. Pseudo‐text is used for all grades in the first level of elementary school. Again, we retained the original text specifications, such as font, size and lining. We followed these specifications by recalculating the proper visual angle.

### 2.3 Procedure

The data were gathered in specially reserved rooms at the Psychological Counselling Centre in (Anonymized city) and at the collaborating elementary school in (Anonymized city), Czech Republic. In both cases, these reserved rooms were properly equipped for regular eye‐tracking measurements. The entire procedure consisted of nine tasks, focusing on both verbal and non‐verbal perception. However, for the purposes of this study, we considered only three reading tasks relevant. These three tasks were administered in the following order: (1) at‐level text, (2) below‐level text and (3) pseudo‐text.

Before the beginning of the experiment, all participants were orally informed about the procedure. The experiment then began with a nine‐point calibration aiming for a maximum deviation of 0.5 visual degrees. The experimental instructions were orally repeated at the beginning of each task, and the experiment proceeded only after the participants confirmed their understanding of the instructions. The main instruction for all three tasks was to read the presented text aloud for 2 min. After this time period, the administrator manually ended the task by pressing the spacebar. To avoid undesirable gaze movements, a gaze‐contingent fixation cross was displayed in the upper left corner before each task.

### 2.4 Apparatus and Eye‐Tracking Features Extraction

All the experimental preparations were carried out using the software Experiment Centre 3.7.69, provided by SMI. The experiments were presented on a 22″ LCD monitor with a screen resolution of 1680 × 1050 pixels and a refresh rate of 60 Hz. The distance between the participants' eyes and the screen was approximately 60–70 cm, ensured using a chinrest to reduce head movements for all participants. Eye movements were captured using an SMI RED 250 remote eye‐tracker with a sampling rate of 250 Hz.

The eye‐tracking data analysis was performed in the licensed SMI software BeGaze v. 3.7. Before analysing the data, we set the high speed‐based saccade event detection to the following parameters: min. duration 22 ms, peak velocity threshold 40°/s, min. fixation duration 50 ms. In the next step, the data cleaning was performed after excluding all the eye‐tracking recordings with a tracking ratio lower than 70%.

For further analysis and classification steps, we exported from BeGaze ‘event data’ files containing the elementary fixations and saccade flow. Event data files relevant for further classification steps contained the eye‐tracking features described in Table 2.

**TABLE 2:** Event data export with relevant eye‐tracking features used for classification steps. (adapted from BeGaze Help Centre n.d.).

Eye‐tracking feature	Description
Participant ID	—
Event start trial time (ms)	Start time of the event (i.e., fixation), relative to the trial start
Event end trial time (ms)	End time of the event (i.e., fixation), relative to the trial start
Event duration (ms)	Total duration of an event (i.e., fixation)
Fixation position *X* and *Y* (px)	Geometric *X* and *Y* position of a fixation on the stimulus; the position of a fixation is calculated as the average of the positions of all samples in that fixation
Fixation dispersion *X* and *Y* (px)	Dispersion of a fixation in the *x*‐direction or *y*‐direction given by [max(*x*) − min(*x*)] or [max(*y*) − min(*y*)]

## 3 Design and Implementation of an Eye‐Tracking and Neural Network System for Dyslexia Detection

Our study introduces INSIGHT, a system designed to detect and classify dyslexia using eye‐tracking data. INSIGHT consists of two core phases. In the first phase, eye‐tracking fixation data are transformed into 2D images, termed ‘Fix‐images’, which offer a clear and intuitive representation of gaze events. These visualisations highlight critical areas where reading difficulties occur, aiding experts in identifying dyslexic patterns. In the second phase, the Fix‐images are analysed using the ResNet18 CNN, a highly effective tool for image classification.

### 3.1 Visualisations

Fix‐images were developed as clear representations of gaze events that aid experts in identifying significant problem areas during the reading process for both non‐dyslexic and dyslexic readers. Each fixation is depicted as an ellipse, with its shape reflecting the dispersion along the *x* and *y* axes. The center of the ellipse corresponds to the average value of *x* and *y* coordinates of the view from both eyes. The colour of the ellipse indicates the fixation duration, with lighter hues indicating longer fixation durations (for further details, refer to Section 2).

**FIGURE 2:**
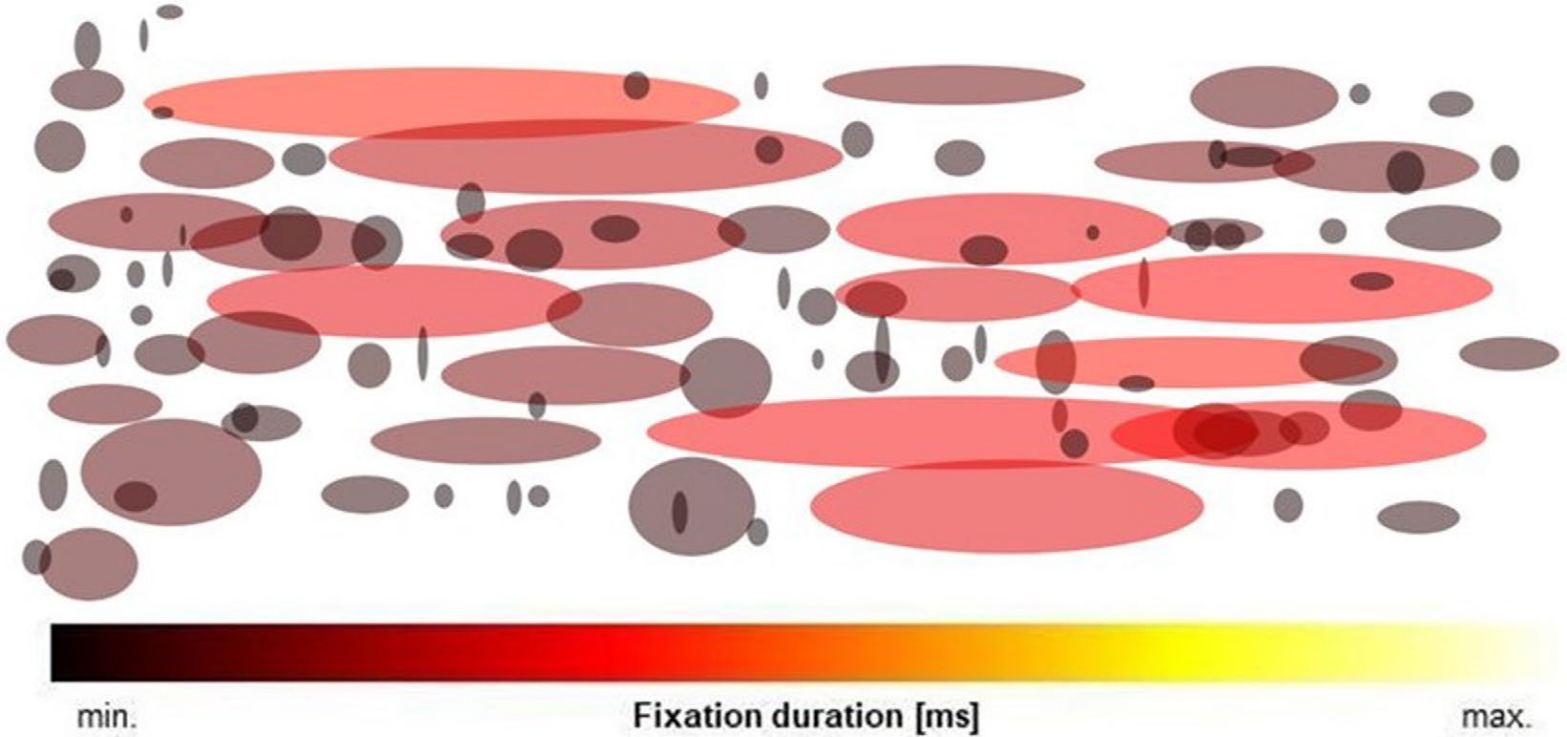
Example of a generated fixation image (Fix‐image).

One of the main contributions of this study is the generation of Fix‐images in relation to a specific task, as illustrated in Figure 2. The generated Fix‐image has a fixed size which is bound by the upper left and lower right corners of the screen area with a displayed text. The Fix‐image basically summarises most of the information about event‐based fixations. In particular, the image consists of ellipses, where each ellipse corresponds to a single fixation—the position of the ellipse corresponds to the fixation center and the radius of the ellipse in *x*/*y* dimensions corresponds to the *fixation dispersion* in the *x*/*y* axes. In other words, the number of visualised ellipses correspond to the number of event‐based fixations. The colour of the ellipse represents the fixation duration normalised for each task independently and ranges from black (very short duration), through red and orange, to yellow (very long duration).

**FIGURE 3:**
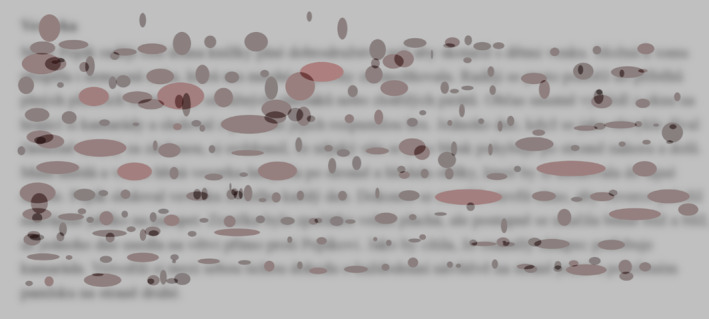
Fix‐image from a control (non‐dyslexic reader) participant reading an at‐level text.

**FIGURE 4:**
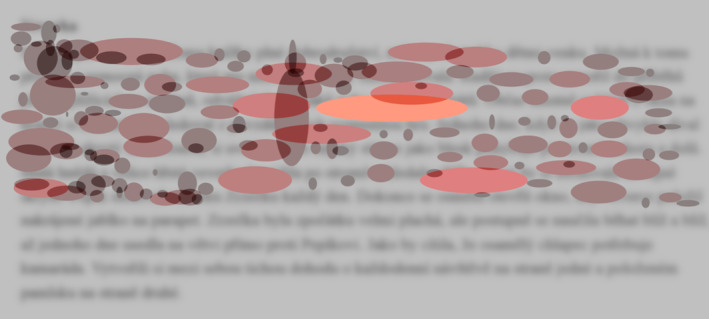
Fix‐image from a dyslexic participant reading an at‐level text.

In Figure 3, we present a Fix‐image visualisation from a non‐dyslexic participant in the fourth grade of elementary school. The selected participant was instructed to read aloud the at‐level text with a specified time limit of 2 min. The Fix‐image shows that the participant was able to read the entire presented text within the given time limit. The underlying text can be observed in this example (the text has been blurred due to copyright protection). In this example, individual line fixations can be observed, including their shape, colour and exact position on the line and on the word. This Fix‐image visualisation enables the fast and simple identification of areas where atypical changes occurred during reading (prolonged fixation duration, etc.). For the selected non‐dyslexic reader, it is evident that the participant processed the text in a fluent manner, as seen in the relatively low number of fixations and their duration. In this Fix‐image visualisation, no obvious difficulties with comprehension or text decoding are evident as would be manifested by prolonged fixations and the ellipsoidal shape of the fixations (for comparison, see Figure 4).

Figure 4 shows a Fix‐image of a dyslexic reader in the 4th grade of elementary school; the unusual reading behaviour is evident. The visualisation shows an at‐level reading text. The dyslexic participant read less than seven lines in a time span of 2 min. The Fix‐image visualisation shows that the dyslexic reader fixated very frequently in specific areas of the text and that the fixation duration was relatively long. The abnormal distribution of individual fixations stands out when compared to the typical fixation dispersion pattern observed in non‐dyslexic readers.

**FIGURE 5:**
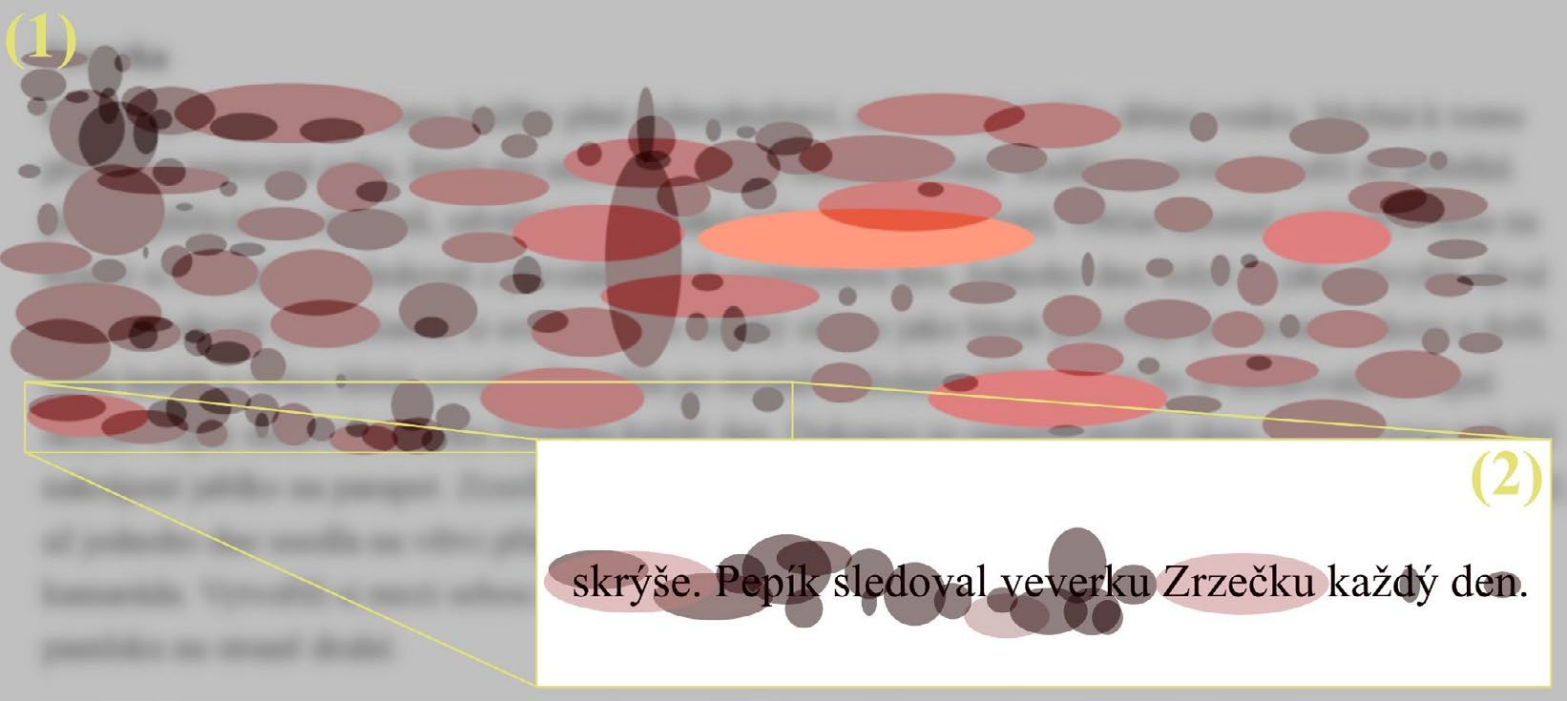
Zoom in for a dyslexic reader.

The Fix‐image visualisation offers a detailed view of individual parts of the text, which can be used to uncover very subtle nuances in reading the text. Zooming in reveals detailed line‐ and word‐level exploration and thus helps experts to detect the sections in the text that were complicated for the reader (e.g., by noting the excessive concentrations of fixations with atypical duration or dispersion, see Figure 5). Such approximations can subsequently lead to the identification of problematic words or text sections for the dyslexic reader and provide experts with cues for the actual diagnostics process and subsequent therapeutic approach selection.

### 3.2 Neural Network‐Based Classification

The main challenge of any deep‐learning classifier is that it generally requires a large number of well‐labelled training data to sufficiently learn the millions of parameters of the underlying neural‐network architecture. In our scenario, only limited event‐based training data were available and the data were complex due to their spatio‐temporal nature. We therefore decided to employ an already pre‐trained CNN *ResNet18* (He et al. 2016) proven to be a successful approach in classifying general‐purpose photos. We slightly change the ResNet18 network architecture and fine‐tune (re‐train) it on the domain of generated Fix‐images to recognise control and dyslexic participants (instead of the original 1000 photo categories).

The first step was to change the last 1000‐dimensional classification network layer (corresponding to 1000 categories of general‐purpose photos) to recognise only two categories—control and dyslexic participants. The second step was to fine‐tune (i.e., additionally re‐train) the network parameters with the generated Fix‐images that correspond to event‐based fixations of available training participants. This second step was performed to increase the classification accuracy. From the technical point of view, the initial size of the generated Fix‐images roughly matches the text as displayed during the reading (~1200 × 400 pixels, depending on the specific task). However, this area is then rescaled to a 224 × 224 RGB array, which is the input size of ResNet18.

**FIGURE 6:**
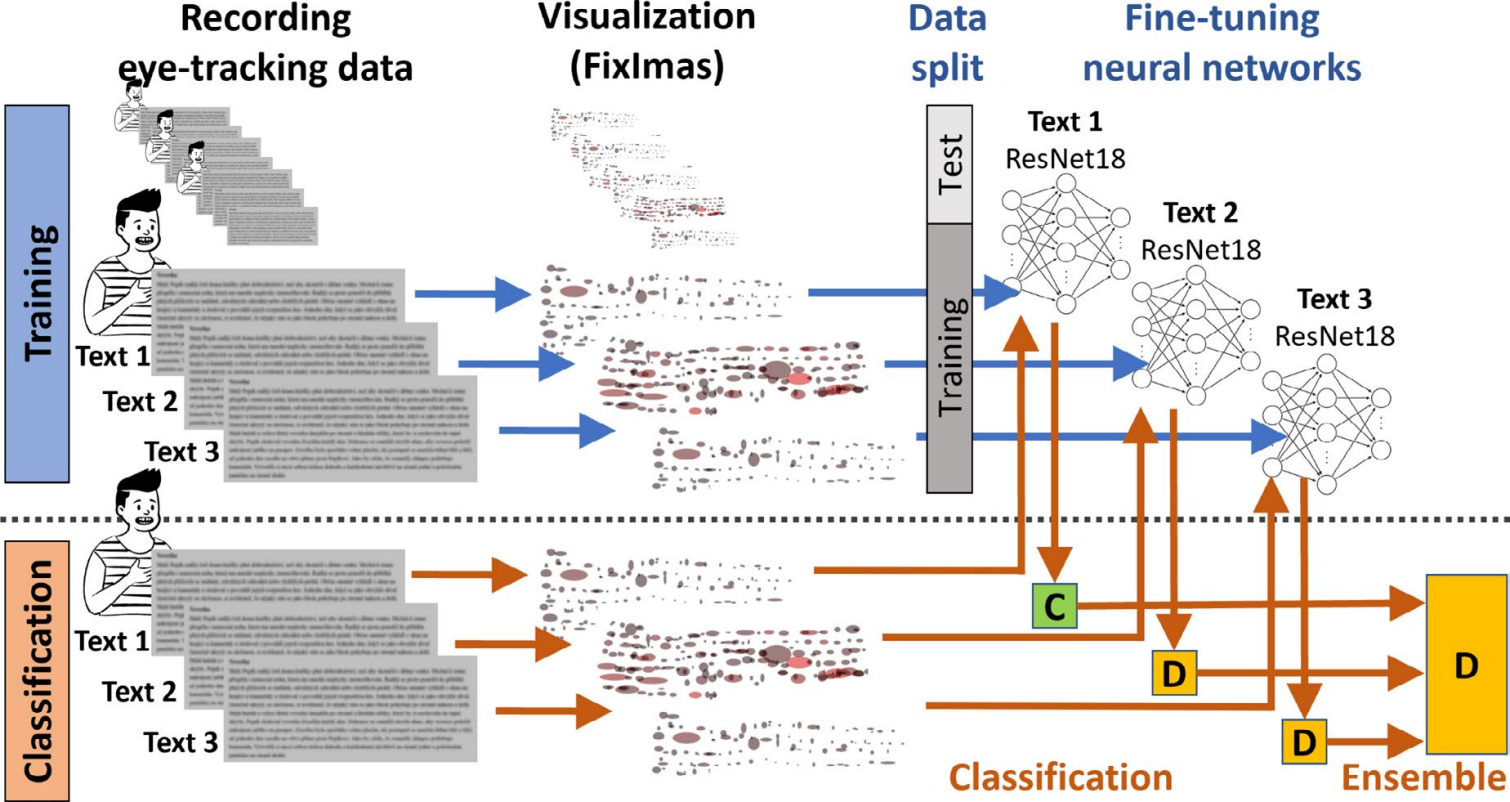
Schematic representation of the whole classification process. The output of each ResNet classifier for a specific task is a binary classification whether the subject is classified as intact C' or dyslexic D'. These partial outputs are aggregated based on majority voting to determine the final subject class (i.e., intact or dyslexic).

The whole classification process for a single text‐reading task is schematically illustrated in Figure 6. As we processed three different tasks, we additionally proposed the ensemble approach to further enhance the classification accuracy. The idea behind the ensemble approach is to combine the classification outputs of the same participant from individual tasks. The most straightforward type of ensemble is majority voting, as successfully applied (e.g., Sedmidubsky and Zezula 2021; Sedmidubsky et al. 2021). In our case of binary classification and an odd number of tasks, we select the class with the highest number of votes.

One of the advantages of the ResNet18 classifier is to provide not only a binary classification (i.e., intact, or dyslexic) but also estimate the probability of how prone an individual is to dyslexia, which is quantified in (%). In this paper, we just simply use the threshold of 50% to determine whether the individual is dyslexic, or not. In this way, we could provide clinicians with such a percentage of tendency to dyslexia, which is more detailed information compared to standard classification. In addition, we can provide this percentage for each task independently (out of three tasks), including the percentage of final majority voting.

#### 3.2.1 Evaluation Methodology

One of the most common pitfalls of machine learning is overfitting: training and evaluating the model on the same data may lead to unfounded confidence in its abilities regardless of its ability to process new instances (Bishop 2006). The solution is to split the data into non‐overlapping training and test sets. However, with small datasets, a small training set can hardly cover the total variability of the real‐world data. To fairly evaluate the available data, we apply the *k*‐fold cross‐validation procedure. This procedure splits the dataset of subjects into *k* distinct folds (sets) and iteratively uses one fold for testing and the rest of *k*–1 folds for training (i.e., fine‐tuning the pre‐trained ResNet18 network), so no mixing between training and test sets occurs. Consequently, there are *k*‐independent experiments so that each dataset subject becomes the test subject to be classified exactly in one experiment—we then report the *mean* classification accuracy over the *k* experiments together with the standard deviation. In particular, we set *k* = 5, which means that 28 subjects are used for training and 7 subjects for testing (out of all 35 dataset subjects). To suppress slight randomness that is inherently included in deep neural network learning, we further evaluate each experiment 10 times (*n* = 10). In this way, *n* × *k* = 50 independent classifiers are trained whose results are finally averaged for each task.

The categorical imbalance (13 dyslexic and 22 control participants) requires another revision. If we used standard *k*‐fold cross‐validation with imbalanced data, we could end up with folds with a test set of a single category (i.e., only intact, or dyslexic subjects). To avoid such imbalanced splits that degenerate the ResNet18 learning process, we stratify the process by separately sampling each category in a balanced way.

We evaluated the 50 experiments for each task by calculating the *balanced accuracy* metric (Brodersen et al. 2010). This metric is convenient when the categories of test data do not contain roughly the same number of examples from each category, which was also the case with our data. The most significant advantage of this metric is that the results are fully comparable with the accuracy of balanced data, i.e., this metric expresses the classification accuracy in the most fair way. This is also the difference from existing papers that commonly use the standard classification accuracy metric even for category‐imbalanced datasets, which can lead to over‐optimistic results.

The balanced accuracy metric is calculated by averaging the recall and specificity for each class. Recall measures the proportion of actual positives correctly identified, while specificity measures the proportion of actual negatives correctly identified. By combining these two values, balanced accuracy provides a comprehensive evaluation that accounts for both the true positive (TP) and true negative (TN) rates, ensuring a more balanced assessment of model performance. Formally, the metric is defined as an average of the TP rate (i.e., recall) and the TN rate (i.e., specificity) as:

$$\begin{array}{l} \text{recall}=\frac{\mathrm{TP}}{\mathrm{TP}+\mathrm{FN}}\\ \text{specificity}=\frac{\mathrm{TN}}{\mathrm{TN}+\mathrm{FP}}\\ \text{balanced accuracy}=\frac{\text{recall}+\text{specificity}}{2} \end{array}$$

## 4 Results of Evaluating the Insight System

In this section, we present the outcomes of three main analyses conducted to evaluate the effectiveness of the INSIGHT system: (1) an analysis of reading performance using machine‐learning classification methods, specifically focusing on the one‐nearest neighbour (1NN) approach applied to selected eye‐tracking features, (2) a neural network‐based evaluation leveraging advanced AI methodologies and (3) a cross‐validation of the proposed AI‐based method on an independent, publicly available dataset from a different language group. The combination of these analyses provides a comprehensive assessment of the INSIGHT system's ability to accurately distinguish between dyslexic and non‐dyslexic readers.

### 4.1 Initial Analysis of Eye‐Tracking Features Using Machine‐Learning Methods

In this chapter, we explore the application of machine‐learning classification methods, specifically focusing on the 1NN classifier, to analyse eye‐tracking data. The goal is to evaluate the contribution of individual features, such as fixation count or total fixation time, in distinguishing between dyslexic and non‐dyslexic readers. Utilising 1NN provides several advantages, including non‐linearity in decision boundaries and adaptability to small datasets, making it an effective preliminary method for understanding feature relevance before delving into more complex AI‐based analyses. This method offers a straightforward, interpretable framework for initial data exploration, ensuring a fair evaluation and establishing a baseline for more advanced techniques. We conduct this analysis as it allows us to comprehensively assess each feature's significance and behaviour independently, helping to validate assumptions and detect any biases early in the research process. This rigorous groundwork is crucial for ensuring that subsequent AI analyses are built on a robust and reliable foundation.

The analysis was performed for all three texts, which were then used for AI‐based analyses. In Table 3, we can observe the results of selected eye‐tracking features recommended by Vajs et al. (2022). We used the 1NN classifier, which is one of the most widely used machine‐learning classification methods in computer science. Specifically, for a given feature value of a particular test subject and task, the closest feature value from a training subject set was identified. The test subject was then classified by the class (i.e., intact, or dyslexic) of the closest training subject, that is, the subject with the most similar value on the particular feature.

**TABLE 3:** Classification accuracy calculated independently for each task based on a 1NN classifier on eye‐tracking features selected by Vajs et al. (2022).

	Below‐level text	At‐level text	Pseudo‐text
Fixation count	61.92 +/− 19.10	71.90 +/− 16.46	45.52 +/− 17.26
Saccade count	48.53 +/− 17.11	64.15 +/− 15.49	68.65 +/− 18.91
Total saccade time (ms)	62.72 +/− 13.49	74.95 +/− 16.15	67.78 +/− 16.04
Total fixation time (ms)	82.03 +/− 14.08	53.87 +/− 18.13	51.67 +/− 13.11
Mean fixation time (ms)	68.55 +/− 13.30	58.28 +/− 15.06	49.77 +/− 15.14

The adoption of the 1NN classifier has several advantages over binary classification based on a single threshold: (1) the decision boundary created by 1NN can generally be highly non‐linear and flexible, (2) 1NN makes decisions based on the closest training example, so it can adapt to various data distributions and shapes of class boundaries, (3) it can perform well even with small datasets, as it directly uses the training data for classification without needing any learning process and (4) it is a non‐parametric and easy‐to‐implement method. Another reason why we adopted 1NN is that we compare the accuracy of 1NN applied not only to a single feature but to the whole time series of fixation data, as described in the next section.

Table 3 presents the classification accuracy for selected eye‐tracking features using the 1NN classifier. The results show considerable variability and generally modest accuracy levels, with percentages ranging from 61.92% for fixation count on below‐level text to 45.52% on pseudo‐text. This considerable variability highlights the limitations of single‐feature analysis with conventional machine‐learning methods, suggesting that these methods may not be adequate for capturing the complex, multi‐dimensional patterns required for accurate dyslexia detection. The relatively low and inconsistent performance across various features underscores the need for more advanced AI techniques that can integrate multiple features and better handle the complexity of the data, leading to improved classification accuracy and more reliable diagnostic outcomes.

### 4.2 Advanced Classification of Dyslexia Using Neural Network‐Based Methods

This section advances the analysis of eye‐tracking data by incorporating neural network‐based methods, notably the fine‐tuned ResNet18 and dynamic time warping (DTW) combined with 1NN. These advanced AI methods allow for the comprehensive representation and classification of time‐series data, capturing the full complexity of eye‐tracking metrics such as fixation positions, durations and dispersions.

Before presenting the actual classification results, we define an additional baseline approach to estimate whether the proposed Fix‐image representation along with the fine‐tuned ResNet18 give reasonable results. Since the Fix‐image representation is generated from the attributes (i.e., center, dispersion and duration) of fixations, we create the time‐series representation based on the same attributes. In particular, we encode each participant‐task experiment into a five‐dimensional sequence (i.e., time‐series), where each sequence item corresponds to the *x*/*y* center, *x*/*y* dispersion and duration of a single fixation. Thus, the sequence length corresponds to the number of fixations and the order of fixations respects their original temporal order. Then, we use the DTW function (Sakoe and Chiba 1978) with the internal Euclidean distance comparing pairs of five‐dimensional fixations, to determine the *similarity* of variable‐length time‐series for two different subjects. DTW is a powerful algorithm that aligns the two sequences by stretching and compressing them to maximise the similarity between their corresponding five‐dimensional fixations, allowing for meaningful comparison even when the sequences vary in length. On top of DTW, we apply the 1NN classifier, similarly as in the previous section where 1NN was applied to a one‐dimensional feature only. In particular, a time series of a test subject is classified based on the label (i.e., intact, or dyslexic) of its mostly similar neighbour in the training set, as determined by DTW. Consequently, 1NN with DTW is effective for time‐series classification tasks, providing interpretable results. In our case, we analyse all the information about fixations (i.e., positions, dispersions, duration, temporal order, etc.) in comparison with other works that extract only simple statistics and classify them using for example, SVMs.

For our analysis, we employed multiple levels: the DTW function for sequence alignment, the 1NN classifier for preliminary classification and the ResNet18 network for deep learning‐based analysis of Fix‐images. These methods were applied to eye‐tracking data across three text types: at‐level text, below‐level text and pseudo‐text. We then computed ensemble summaries to provide an integrated view of the classification performance.

The 1NN‐DTW method showed promising results, particularly with the at‐level text, achieving 83.25% accuracy. However, accuracy decreased for below‐level text (75.55%) and pseudo‐text (73.82%), resulting in an overall ensemble accuracy of 80.78%. Incorporating fixation dispersion on the *x* and *y* axes in the DTW model (1NN‐DTW *XYL*DxDy) slightly improved the overall ensemble accuracy to 81.33%, although it led to a decrease in the at‐level text accuracy (81.90%).

The ResNet18 method outperformed the DTW‐based methods, demonstrating superior accuracy across all text levels, particularly for at‐level text (88.78%), indicating its efficacy in detecting subtle differences in eye movements between dyslexic and non‐dyslexic readers. There was also an increase for below‐level text and pseudo‐text (see Table 4). The Fix‐image representation, combined with ResNet18, achieved an overall ensemble accuracy of 86.65%, highlighting the potential of deep learning techniques in capturing complex patterns in eye‐tracking data. The relatively lower performance of the DTW‐based 1NN method across the three tasks underscores the need for more sophisticated neural network methods to achieve higher accuracy and reliability in classification tasks.

**TABLE 4:** Results of the 1NN‐DTW and ResNet18 methods.

w/all samples	Below‐level text	At‐level text	Pseudo‐text	ensemble
1NN‐DTW *XYL*	75.55% +/− 17.04	83.25% +/− 14.09	73.82% +/− 16.67	80.78% +/− 16.04
1NN‐DTW *XYL*DxDy	76.85% +/− 14.94	81.90% +/− 14.20	72.15% +/− 16.45	81.33% +/− 15.06
Fix‐image + ResNet18	78.40% +/− 14.23	88.78% +/− 13.18	74.13% +/− 17.03	86.65% +/− 13.24

*Note: X* and *Y* indicate gaze position, L indicates fixation duration, and Dx/Dy describes the dispersion of the fixation on *x* and *y* axes. Symbol +/− is used to signify the standard deviation of the experiment results.

### 4.3 Explainability

One of the primary challenges in developing and deploying deep neural networks is explainability, which involves understanding the model's rationale for any given classification. Such understanding could aid the clinician in verifying the diagnosis or make it easier to correct it if it is wrong (Kasprowski 2024).

For CNNs, such as ResNet18, trained on RGB images, most explainable AI techniques focus on generating saliency maps, which highlight the important parts of the input images. An example is Grad‐CAM, which identifies critical regions from the perspective of a selected layer, such as the final convolutional layer.

We employed a model trained on a less accurate test fold to display all combinations of true and predicted labels with respect to the last convolutional layer, chosen for its readability, and the predicted class. The results indicated that Fix‐images predicted as dyslexic had the network's attention concentrated on smaller regions. Conversely, non‐dyslexic predictions showed a more widespread focus over the white space. These findings suggest that a model trained on a larger dataset could better delineate the areas of Fix‐images that clinicians should prioritise.

Table 5 illustrates the critical regions in Fix‐images, as determined by true and predicted labels for dyslexic and non‐dyslexic classifications. The saliency maps indicate the model's focused attention in accurately classified dyslexic cases, reflecting sensitivity to specific features. In contrast, the focus in non‐dyslexic predictions is more diffused, implying less specificity. These observations highlight the potential of explainable AI to enhance the transparency and reliability of models in clinical diagnostics, thereby aiding clinicians and enhancing the precision of dyslexia detection.

**TABLE 5:** Confusion matrix showing important areas of the input Fix‐images.

		True	
		Dyslexic	Non‐dyslexic
Predicted	Dyslexic	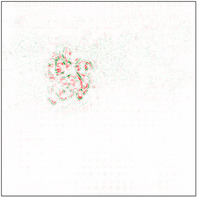	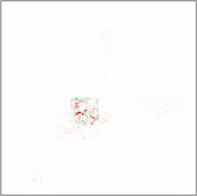
	Non‐dyslexic	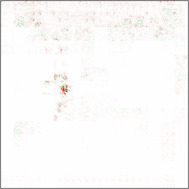	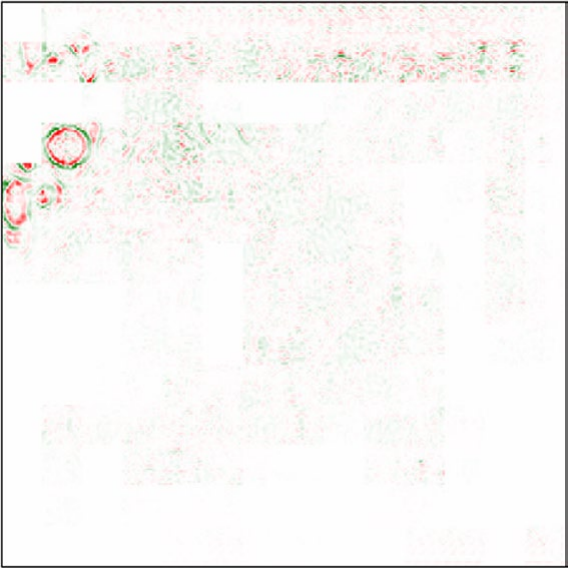

### 4.4 Cross‐Linguistic Validation of the INSIGHT Method on an Independent Eye‐Tracking Dataset

To test generalizability across populations and languages, we validated our method on publicly available eye‐tracking datasets of dyslexic and non‐dyslexic readers. We have followed three core criteria to select the relevant dataset: First, we considered the technical aspects of the datasets which include public availability of the dataset and also the suitable eye‐tracking parameters, such as data collection performed using a remote eye‐tracking device and proper eye‐tracking features for further analyses. Second, the aspect of population and language diversity was crucial. For this, we considered datasets containing a wide age range of participants and, due to the linguistic similarities across Slavic languages, we focused on a language from a different language family. Finally, to consider the selected dataset reliable, we also requested that such a dataset had already been used for analyses in other research studies.

Based on these criterions, we have chosen the state‐of‐the‐art CopCo dataset (Hollenstein, Barrett, and Björnsdóttir 2022), an eye‐tracking corpus involving 57 Danish readers (aged 20–64), from which we selected 18 dyslexic and 18 non‐dyslexic participants to match the existing research on the dataset for the comparative experiments. Each participant read a random selection of texts, with only one text shared among all of them. This text was further split into individual trials, paragraphs that were shown sequentially, separated by synchronisation phase. From nine trials on the shared text, we chose six that contained multiple lines to have as much data as possible.

From the fixation data exported by the dataset authors, we have generated the Fix‐images. However, since the data did not include fixation dispersion, we have used constant ellipse size. We used a similar methodology as on our data but using the nine‐fold cross validation to also match the state‐of‐the‐art and training separate model for each trial. Furthermore, we also produced ensemble summaries (using five random trials) to stabilise the results.

Kasprowski (2024) trained a CNN on an alternative reading visualisations of this dataset and reports the best accuracy of around 78% (standard deviation not available) with respect to a comparable evaluation scenario (using the classification threshold of 50% as we did). Our ensemble significantly outperforms this with the accuracy of 86.11% +/− 12.42, and on one trial, we even achieved the accuracy of 94.44% +/− 10.39.

## 5 Discussion

In this study, we aimed to present a novel and advanced AI‐based approach, the INSIGHT method, for detecting reading disabilities in children. The suggested AI approach is based on two steps. In the first phase, we propose a visualisation method based on fixation events of the gaze‐recorded data called Fix‐images for further neural network analysis. Fix‐images can outline dyslexic readers' reading abilities while reading a text in a clear and simple way, including the possibility of zoomed visualisation at sentence and word levels. The Fix‐image visualisation method may offer diagnostic experts valuable information about the overall reading process of a dyslexic child with the possibility to zoom in to observe atypical reading fixation events on a line‐and‐word level. Fix‐images visualisations have additional evident potential beyond aiding in the diagnostics of dyslexia. With the assistance of these visualisations it is possible to clearly detect places and areas in text where a dyslexic has reading difficulties (see Figures 4 and 5), such visualisations may be assistive for example, in the context text adaptation, such as lexical or graphical simplification as part of easy‐to‐read guidelines (see e.g., Rivero‐Contreras, Engelhardt, and Saldaña 2021), which could make the text more feasible for dyslexics to work with a textual information processing.

Compared to current gaze recording analysis applications (e.g., BeGaze v. 3.7 n.d.), our visualisation method provides a simple and clear representation of several core features: fixation position, fixation duration, and specifically fixation dispersion, which is absent in conventional analysis software. In the second phase, the Fix‐images are used for advanced neural network level analysis, which provides a general evaluation of the reading performance with relatively high accuracy.

Our results show that the at‐level text offers the highest classification potential for detecting dyslexic readers, reaching 88.78% classification accuracy. The use of at‐level text has been applied by other researchers, for example, Nilsson Benfatto et al. (2016), Vajs et al. (2022), and Rello and Ballesteros (2015) or Sedmidubsky et al. (2025), who interpreted higher levels of classification accuracy.

Considering the below‐level text, the results of the classification process are considerably lower than with the at‐level text. With the below‐level text, the classification level decreased to 78.40% accuracy. This decreasing pattern was also reported by Smyrnakis et al. (2017), who applied a similar methodological approach, presenting participants with two levels of text stimuli: the first version of the text was set to the same age level of the participants, the second version was then set to a below age level text. Smyrnakis et al. (2017) showed an effect similar to our results: it is apparent that the below‐level text is easier for dyslexic readers to read and dyslexic gaze patterns appear to be reduced. This pattern was also evident in Trauzettel‐Klosinski et al. (2010), though the authors do not apply an AI approach nor visualisations for the data analysis.

The performance of the pseudo‐text reading is comparable with the below‐level text in terms of its potential for dyslexia detection. The application of pseudo‐text for AI‐based classification purposes is novel in the field of automatized dyslexia detection based on gaze data. Although there is not much research in the area of pseudo‐text reading, this type of text is usually used in the Czech environment to diagnose specific learning disabilities (see Bednářová 2015). For this reason, we decided to use this type of text in the study. Research in pseudo‐word reading (e.g., Hutzler and Wimmer 2004) has shown that the differences in eye movements between the dyslexic and regular readers are significant; however, they do not appear to be highly salient for AI classification purposes as the classification accuracy reached only 74.13%. This effect may be due to several factors, such as reduced word predictability of the non‐dyslexic readers, which eventually can lead to a reduction in overall reading speed and fluency. Further research should be conducted in this area. Additionally, also worth mentioning is the contribution of Rayner (1998), which is based on studies from the 1980s and 1990s. Some of our results, for example the difference between dyslexic and intact readers in fixation time, saccade length or number of fixations (see the Fix‐images visualisation) support these statements. A correlation is also evident during reading of below‐level and at‐level text (see Table 4), which, according to Rayner (1998), may be related to parafoveal processing of text during fixations.

From the machine learning point of view, there are several advantages of the proposed approach: (1) universality—raw eye‐tracking data, as well as event‐based features (e.g., fixations or even saccades), can be quite easily transformed into a 2D Fix‐image without the loss of eye‐tracking information, (2) rapidly evolving research in computer science continually provides more and more effective neural networks for the classification of image data, so it is possible to test different kinds of architectures on the domain of Fix‐images in the future (we adopted the ResNet18 classifier as it reaches a reasonable balance between classification accuracy and training complexity), (3) ResNet18 does not require many training examples as we only fine‐tune the pre‐trained ResNet18 version on the domain of general‐purpose photos; this is probably the biggest advantage since other machine learning methods, such as SVM or deep neural networks, typically require more training data to be trained from the scratch. The proposed approach leads to noticeable improvements over the machine‐learning baseline, and the mean accuracy of 86% already reaches the top levels that can be achieved without overfitting. Notice that designing and training a custom network architecture would not be reasonably feasible due to a small training dataset.

The INSIGHT method proved to be an effective tool for detecting and classifying dyslexic readers based on eye‐movement recordings. This method can be of considerable help to diagnostic experts, as it provides them with complementary rapid and insightful information about the dyslexic readers reading patterns, through a simple Fix‐image visualisation, which is subsequently usable for AI‐based evaluation.

## 6 Conclusion

In this study, we presented a new approach called INSIGHT, aiming at dyslexia detection. Our INSIGHT method combines state‐of‐the‐art research on dyslexia with current technological advances and is based on two phases: visualisation and subsequent neural network based classification. This method offers diagnosticians of specific learning disabilities an alternative view of the objective examination screening of a child's reading performance based on the gaze data recorded during the reading process. This innovative approach merges two key elements: providing experts with a transparent view of gaze data not typically included in the diagnostic process, and delivering an AI‐based evaluation of overall reading performance. This integration has the potential to significantly enhance the entire process of screening and diagnosing dyslexia, enabling faster and more precise assessments and the subsequent selection of appropriate therapeutic interventions.

Future research and development could explore several promising directions. It would be beneficial to focus on individual aspects of dyslexia, consider the severity level of the condition and evaluate the applicability of this method across different languages. Methodologically, enhancing the accuracy of classification could involve: (1) augmenting Fix‐image visualisations to artificially expand the training dataset, thereby aiding the ResNet18 network in learning more complex relationships among fixation data; (2) experimenting with other pre‐trained image‐based classification networks, such as ResNet50 or Vision Transformers (Dosovitskiy et al. 2021); or (3) employing entirely different neural network architectures, like RNNs, which have shown success in modelling multi‐dimensional time‐series data, such as human motion (Sedmidubsky and Zezula 2021; Sedmidubsky et al. 2021).

The INSIGHT method holds promise for implementation in psychological counselling centers as a supplementary tool for dyslexia diagnosis. It is simple to administer using a portable eye‐tracking device, which involves presenting three short texts and interpreting the resulting data. This accessibility makes it a practical option for widespread use in diagnostic settings.

## Ethics Statement

The Research Ethics Committee of Masaryk University approved the research plan and its implementation.

## Conflicts of Interest

The authors declare no conflicts of interest.

## Data Availability

The data that support the findings of this study are available from the corresponding author upon reasonable request.
